# Modeling the developmental origins of pediatric cancer to improve patient outcomes

**DOI:** 10.1242/dmm.048930

**Published:** 2021-02-22

**Authors:** James F. Amatruda

**Affiliations:** Cancer and Blood Disease Institute, Children's Hospital Los Angeles, Los Angeles, CA 90027, USA

## Abstract

In the treatment of children and adolescents with cancer, multimodal approaches combining surgery, chemotherapy and radiation can cure most patients, but may cause lifelong health problems in survivors. Current therapies only modestly reflect increased knowledge about the molecular mechanisms of these cancers. Advances in next-generation sequencing have provided unprecedented cataloging of genetic aberrations in tumors, but understanding how these genetic changes drive cellular transformation, and how they can be effectively targeted, will require multidisciplinary collaboration and preclinical models that are truly representative of the *in vivo* environment. Here, I discuss some of the key challenges in pediatric cancer from my perspective as a physician-scientist, and touch on some promising new approaches that have the potential to transform our understanding of these diseases.

## Abstract (precis)

**Summary:** This Perspective discusses the special features that make it challenging to develop new therapies for pediatric cancers, and the ways in which collaboration centered on improved models can meet these challenges.

## Introduction: the challenge of pediatric cancer

Cancer in children makes up ∼3% of the global incidence of cancer, translating to ∼16,500 new cases/year in the United States. Despite these relatively small numbers, pediatric cancer exerts an outsized impact: beyond the physical and emotional toll on children, their families and the community, the number of years of productive life lost to pediatric cancers is proportionately higher than that of adult cancers. While there is much to celebrate in the treatment of pediatric cancers – overall survival rates for children with cancer have improved from less than 10% in the 1960s to around 80% today – it is fair to say that much of this improvement had little to do with better understanding of tumor biology. Instead, progress has largely been based on the empiric use of multimodality therapy (surgery, chemotherapy, radiation) that still serves as first-line treatment for the vast majority of pediatric cancer patients. The cost of this success becomes more and more apparent each year, as the growing number of survivors of childhood cancer face lifelong adverse health effects due to the toxicity of chemotherapy and radiation (Suh et al., 2020). Recognition of the problems associated with non-targeted therapies has spurred efforts to develop alternative approaches for childhood cancers that could be more effective and less toxic than current treatments.

“The cost of [pediatric cancer] success becomes more and more apparent each year, as the growing number of survivors of childhood cancer face lifelong adverse health effects due to the toxicity of chemotherapy and radiation.”

Advances in next-generation sequencing have made possible one such approach, known as precision medicine. This strategy is based on genomic profiling of a given patient's tumor, yielding information that can then be used to select therapies designed to counter the effects of specific driver mutations. The approach has had some notable successes, including the treatment of pediatric cancers with *NTRK* or *ALK* gene rearrangements (Butrynski et al., 2010; Drilon et al., 2018; Laetsch et al., 2018). In addition to genomics, a lot of progress has been made in the regulatory environment and the pharmaceutical industry, enabling cooperative trials of precision medicine, such as Pediatric Molecular Analysis for Therapy Choice (MATCH) in the USA, Precision Oncology for Young People (PROFYLE) in Canada and Individualized Therapy for Relapsed Malignancies in Childhood (INFORM) in Europe (Hadjadj et al., 2020). One of the largest such studies to date, the Zero Childhood Cancer (ZERO) initiative in Australia, illustrates both the promise and challenges of the precision medicine approach. In this study, a combination of whole-genome sequencing and RNA sequencing was used to analyze more than 250 tumor specimens, resulting in specific therapeutic recommendations for two-thirds of the patients (Wong et al., 2020). Of 38 evaluable patients in this group with high-risk cancers, 31% exhibited a complete or partial response, comparable to results from larger precision medicine trials in adults (Hazim and Prasad, 2018). While encouraging, the long-term benefits of this ‘sequence tumor, choose agent’ paradigm may ultimately apply only for a small percentage of patients (Marquart et al., 2018). The reasons for the lower impact are several, but accumulating evidence suggests that one major challenge is the difficulty of predicting the biological impact of specific mutations and the efficacy of targeted therapy from genomic data. This is almost certainly due to the complex nature of tumor cell behavior in the *in vivo* environment, where tumor cell growth, survival and treatment response may depend on multiple genetic and epigenetic features of both cancer cells and normal host cells in the tumor microenvironment. It is likely that, in children, special features of pediatric cancers will create even more difficulties for the precision medicine approach. Below, I discuss these features, as well as some potential strategies to improve the effectiveness of targeted therapies for childhood cancer.

The most common cancers of adults are carcinomas of the lung, breast, colon, prostate and other epithelial tissues; however, these cancers are vanishingly rare in children. Instead, acute leukemias and brain tumors predominate. Another major category of childhood cancer is the so-called embryonal tumors or blastomas, such as neuroblastoma, medulloblastoma, nephroblastoma and hepatoblastoma. This category of tumors, which also includes germ cell tumors and certain sarcomas, is striking because of the resemblance of the tumor cells to the corresponding fetal tissue, albeit with features of aberrant development (Chen et al., 2015; Gojo et al., 2020; Scotting et al., 2005). These histologic clues to the developmental origins of pediatric cancers are supported by several other features. The unique age spectrum associated with most childhood cancers suggests that there are time- and tissue-specific windows of susceptibility to cell transformation (Johnston et al., 2020; Linabery and Ross, 2008). Pediatric cancers exhibit fewer somatic mutations on average than adult cancers (Grobner et al., 2018), and some are driven by features such as transcription factor fusion oncogenes, which have so far been refractory to targeting. Pediatric cancers also disproportionately carry alterations in epigenetic factors (Huether et al., 2014) and developmental signaling pathways such as WNT, Notch, TGF-beta and Hedgehog (Filbin and Monje, 2019), which can represent novel targets but also introduce therapeutic complexity owing to the importance of these factors in normal, developing tissues in children (Chheda and Gutmann, 2017; Gajjar et al., 2013; Morinello et al., 2014; Zwergel et al., 2018). The theme of early development is further apparent in the strong linkage between cancer predisposition and developmental syndromes, such as Noonan and Costello RASopathy syndromes, or overgrowth syndromes such as Beckwith-Wiedemann or Perlman's syndromes (Bharathavikru and Hastie, 2018; Cizmarova et al., 2013). Pediatric cancers arise during a time period of profound changes in tissue patterning and organ development. Any attempt to fully understand the origin of these cancers must therefore take into account not only the spectrum of molecular lesions linked to specific cancer types, but also the particular biological features and developmental stage of the tissue lineage in which the cancers arise.

## Modeling developmental mechanisms of childhood cancer

How can developmental biology contribute to better understanding of tumor biology and ultimately to better outcomes for children with cancer? How do we move beyond *in silico* analyses or studies done with cell lines grown on plastic? Certainly xenografts, especially orthotopic and patient-derived xenografts, hold promise as potentially more accurate models (Aparicio et al., 2015; Hermans and Hulleman, 2020; Zarzosa et al., 2017), although the overall rarity of most pediatric cancers can make it difficult to assemble large cohorts. *In vitro*, innovative spheroid culture approaches have begun to reveal dramatic changes in drug sensitivity of the same cells cultured in three-dimensional versus two-dimensional environments (Breslin and O'Driscoll, 2016; Fujii et al., 2009; Imamura et al., 2015; Musah-Eroje and Watson, 2019; Polo et al., 2010). Organoid models – self-organizing tissues grown *in vitro* from stem or progenitor cells, exhibiting lineage-specific cell differentiation and the formation of tissue architecture resembling the relevant organ – can provide great advantages for live imaging (Bolhaqueiro et al., 2018; Srivastava et al., 2020), metabolic studies (Browne et al., 2017) and drug screening (Burkhart et al., 2018; Francies et al., 2019; Jabs et al., 2017). Many pediatric cancers are thought to arise during embryonic or fetal life, meaning that the relevant stem and progenitor cell populations are often unknown or difficult to obtain, complicating efforts to build relevant organoid models. One approach to overcome this barrier involves the generation of induced pluripotent stem cells (iPSCs), which can then be directed to differentiate along lineage-specific trajectories. Introducing oncogenic mutations into the cells, or deriving the iPSCs from donors carrying germline cancer susceptibility mutations, provides a source of progenitors that can be developed into cancer organoids or established as xenografts in immunocompromised mice (Dost et al., 2020; Hwang et al., 2019; Papapetrou, 2016). This method has produced models of pediatric cancers including medulloblastoma (Huang et al., 2019) and retinoblastoma (Saengwimol et al., 2018). For some cancers, such as hepatoblastoma (Saltsman et al., 2020) and Wilms tumor of the kidney (Calandrini et al., 2020), tumor-derived cells have been used to develop organoids exhibiting multilineage potential.

**Figure:**
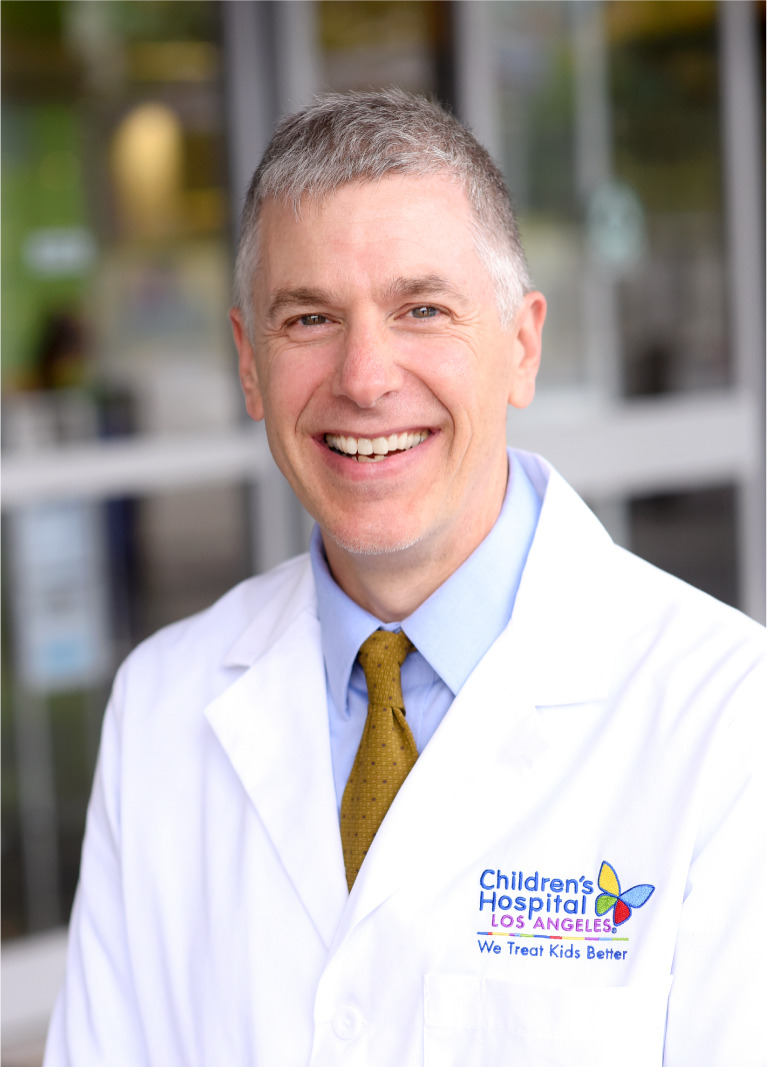
**James F. Amatruda, MD, PhD.** Head of Basic and Translational Research in the Cancer and Blood Disease Institute and the Division of Pediatric Hematology-Oncology at Children's Hospital Los Angeles; and Professor of Pediatrics and Medicine at the Keck School of Medicine of the University of Southern California.

Model organisms can contribute greatly to understanding cancer pathways, even without directly modeling tumorigenesis. For example, studies of the effects of gene regulatory networks and signaling pathways on early embryonic development have provided critical insight into cancer-relevant phenomena including RAS signaling (Beitel et al., 1990; Han and Sternberg, 1990; Simon et al., 1991), microRNA biology (Lee et al., 1993; Wightman et al., 1993) and programmed cell death (Yuan and Horvitz, 2004). Mutations in mammalian oncogenes and tumor suppressors, when engineered into model organisms, may cause developmental phenotypes that can be used as a platform for testing small molecules or genetic modifiers (Al-Olabi et al., 2018; Anastasaki et al., 2012; Levinson and Cagan, 2016; Liu et al., 2017; van der Hoeven et al., 2020; Vidal et al., 2005).

Further insight can be provided by models that attempt to recapitulate human cancer genetics via regulated expression of gain- and loss-of-function cancer mutations in the relevant organ or tissue. Such models, instituted in genetically engineered mice, worms, fish, flies and other organisms, have perhaps the greatest potential to exhibit the full range of cancer phenotypes encountered in patients, including the role of germline (inherited) variants, tumor initiation and growth, the role of the tumor microenvironment, interaction with the host immune system, metastasis and response to treatment. There are many examples pointing to the success of this strategy for modeling lung cancer, brain tumors, breast cancer, melanoma, leukemias, testicular cancer and other malignancies (Annunziato et al., 2016; Ceol et al., 2011; Johnson et al., 2001; Kohnken et al., 2017; Meuwissen et al., 2003; Milagre et al., 2010; Patton et al., 2005; Pierpont et al., 2017; Read et al., 2009).

In the case of pediatric cancers, the ‘cell-of-origin’ problem discussed above presents a challenge for models, requiring investigators to choose carefully when targeting expression of candidate cancer-driving genetic changes to specific tissues. Here is where model organisms can be especially useful, as they can provide access to a range of developmental time windows and tissue lineages, as well as reflect the changing epigenetic landscape of early development. Targeting these developmental compartments has allowed the generation of animal models of neuroblastoma, rhabdomyosarcoma, medulloblastoma and Wilms tumor, among other childhood cancers (Hackett et al., 2003; Keller et al., 2004; Kendall et al., 2018; Stewart et al., 2010; Urbach et al., 2014; Zurawel et al., 2000). The value of model organisms is especially clear in the case of pediatric brain tumors, where molecular profiling has increasingly revealed distinct epigenetically defined subgroups within tumor types, with important clinical implications. For example, single-cell analysis of tumors, coupled with cross-species transcriptomics of mouse neural development, provides new insight into the distinct developmental origins of medulloblastoma subtypes (Hovestadt et al., 2019; Marino and Gilbertson, 2021). And in a recent review in Disease Models & Mechanisms, Cédric Maurange elegantly lays out the case that fundamental work on temporal patterning in *Drosophila* neural progenitors can inform our understanding of the origins of a range of pediatric brain tumors (Maurange, 2020).

“[…] model organisms can be especially useful, as they can provide access to a range of developmental time windows and tissue lineages, as well as reflect the changing epigenetic landscape of early development.”

In parallel to these efforts in worms, flies and mice, we and others have turned to zebrafish as a powerful and flexible animal model for human cancer. The strengths of the fish model for genetic modeling, imaging and drug screening have previously been described (Amatruda and Patton, 2008; Casey and Stewart, 2020; Mayrhofer and Mione, 2016; Xie et al., 2015; Yen et al., 2014). In the context of pediatric cancer, one of the most valuable aspects of the fish model is the access it provides to a range of developmental time windows and tissue lineages, some only present during early development and absent at the adult stage. Pioneering work modeling T-cell leukemias in fish (Langenau et al., 2003) enabled use of the system to discover novel leukemia genes such as *ARID5B* (Leong et al., 2017) and *JDP2* (Mansour et al., 2018). A transgenic model of neuroblastoma (Zhu et al., 2012) highlighted the role of developmental apoptosis as an oncogene-induced antitumor response. Models of central nervous system primitive neuroectodermal tumor (PNET) identified oligodendrocyte precursor cells as a cell of origin for this pediatric brain tumor subtype, and provide a platform for drug testing (Modzelewska et al., 2016). Zebrafish melanoma models have probed links between RAS signaling, development and cancer (Anastasaki et al., 2009; Patton et al., 2005), and have identified reactivation of embryonic developmental pathways as a critical event in tumor initiation (Kaufman et al., 2016; White et al., 2011). The role of epigenetic modifiers has been tested in fish models of liver cancer, myelodysplastic syndrome and rhabdomyosarcoma (Albacker et al., 2013; Chernyavskaya et al., 2016; Gjini et al., 2015; Mudbhary et al., 2014). Zebrafish modeling RAS-driven embryonal rhabdomyosarcoma have elegantly probed the cell of origin of this disease (Storer et al., 2013; Tenente et al., 2017). Our own work on alveolar rhabdomyosarcoma, a clinically aggressive tumor driven by oncogenic PAX3–FOXO1 fusion proteins, leveraged developmental assays in zebrafish embryos and adult tumor models to identify *HES3* as a cooperating oncogene that impairs muscle differentiation and contributes to poor clinical outcomes (Kendall et al., 2018). Collectively, these studies demonstrate the power of applying a developmental biology approach to generate key insights into the origin and uncontrolled growth of pediatric cancers.

“[…] integrated projects will accommodate multiple scales of speed – including both the slow, meticulous process of building, evaluating and refining models, and in parallel the rapid generation of custom models reflecting an individual patient's genetics, with real-time return of results to the treatment team […]”

## Looking forward

**Fig. 1.**
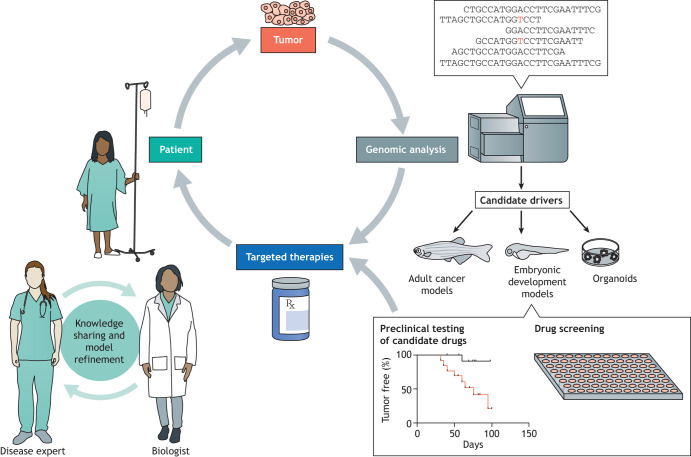
**Improving therapy of pediatric cancers through collaboration.** Next-generation sequencing of tumor samples may directly identify candidate targeted therapies. In many cases, further investigation is required. Model systems such as organoids or genetically engineered animals can interrogate the function of candidate driver genes in a setting that recapitulates the complexity of the *in vivo* tumor environment. Such models can support drug screening and preclinical testing of novel therapies. Throughout, collaboration between clinicians and basic scientists is essential to define clinical challenges and to build and refine disease models.

Today, while it is true that most cancers are still treated with combinations of surgery, radiation therapy and chemotherapy, important progress in the development of molecularly targeted and immune-modulating therapies has begun to change this paradigm, albeit slightly. To continue and expand on this progress will take a concerted effort, one in which mechanistically based models will play a crucial role (Fig. 1). Developmental biology, by its nature concerned with understanding gene function in the fuller context of tissue lineage and cell–cell interactions at the organism level, has a lot to contribute to the understanding of how genomic and signaling alterations lead to unrestrained growth of pediatric cancers. To achieve this, more powerful understanding will require new types of collaborations between the cancer and developmental biologists who build these models with pathologists, oncologists and other disease experts. This team approach can serve, through iterative feedback and discussion, not only to define the clinically important problems, but also to ‘credential’ a given model as representative of the human disease – especially important, as we must acknowledge the inherent limitations of all of our models. Ideally these integrated projects will accommodate multiple scales of speed – including both the slow, meticulous process of building, evaluating and refining models, and in parallel the rapid generation of custom models reflecting an individual patient's genetics, with real-time return of results to the treatment team in a time window that can benefit that patient. A recent inspiring example is one in which a zebrafish model of a novel gene variant suspected of causing lymphatic anomaly led to successful treatment of the patient (Li et al., 2019). One can imagine a similar approach being applied for pediatric cancers, including clarifying the pathogenic role of variants of unknown significance.

In this effort, it will be especially important for new models to reflect the great heterogeneity of human disease processes, not only intratumoral heterogeneity, but also the effects of gender, race and ethnicity, which may strongly impact disease phenotypes and response to treatment. Involvement of patients and advocates as members of these interdisciplinary teams can further help to prioritize research goals. Moving forward will require not only collaboration but also creativity, finding new ways to recognize the efforts of team members with diverse skillsets, and sustaining funding for disease-focused research without neglecting the fundamental importance of basic research in molecular and developmental biology. While much work is still required to address therapy resistance and metastasis, we may look forward to bringing the formidable power of molecular developmental biology to bear for the benefit of children with cancer and other diseases.
